# Evaluation in medical education: A topical review of target parameters, data collection tools and confounding factors

**DOI:** 10.3205/000219

**Published:** 2015-09-16

**Authors:** Sarah Schiekirka, Markus A. Feufel, Christoph Herrmann-Lingen, Tobias Raupach

**Affiliations:** 1 Universitätsmedizin Göttingen, Studiendekanat, Göttingen, Germany; 2 Charité – Universitätsmedizin Berlin, Prodekanat für Studium und Lehre, Berlin, Germany; 3 Max-Planck-Institut für Bildungsforschung, Forschungsbereich Adaptives Verhalten und Kognition und Harding Zentrum für Risikokommunikation, Berlin, Germany; 4 Universitätsmedizin Göttingen, Klinik für Psychosomatische Medizin und Psychotherapie, Göttingen, Germany; 5 Arbeitsgemeinschaft der Wissenschaftlichen Medizinischen Fachgesellschaften, Düsseldorf, Germany; 6 Universitätsmedizin Göttingen, Klinik für Kardiologie und Pneumologie, Göttingen, Germany; 7 University College London, Health Behaviour Research Centre, London, Great Britain

**Keywords:** evaluation, medical education, dimension, confounder, questionnaire

## Abstract

**Background and objective:** Evaluation is an integral part of education in German medical schools. According to the quality standards set by the German Society for Evaluation, evaluation tools must provide an accurate and fair appraisal of teaching quality. Thus, data collection tools must be highly reliable and valid. This review summarises the current literature on evaluation of medical education with regard to the possible dimensions of teaching quality, the psychometric properties of survey instruments and potential confounding factors.

**Methods:** We searched Pubmed, PsycINFO and PSYNDEX for literature on evaluation in medical education and included studies published up until June 30, 2011 as well as articles identified in the “grey literature”. Results are presented as a narrative review.

**Results:** We identified four dimensions of teaching quality: structure, process, teacher characteristics, and outcome. Student ratings are predominantly used to address the first three dimensions, and a number of reliable tools are available for this purpose. However, potential confounders of student ratings pose a threat to the validity of these instruments. Outcome is usually operationalised in terms of student performance on examinations, but methodological problems may limit the usability of these data for evaluation purposes. In addition, not all examinations at German medical schools meet current quality standards.

**Conclusion:** The choice of tools for evaluating medical education should be guided by the dimension that is targeted by the evaluation. Likewise, evaluation results can only be interpreted within the context of the construct addressed by the data collection tool that was used as well as its specific confounding factors.

## Zusammenfassung

**Hintergrund und Fragestellung:** Die Evaluation ist fester Bestandteil der Lehre an Medizinischen Fakultäten. Gemäß den Standards der Deutschen Gesellschaft für Evaluation müssen Evaluationsinstrumente eine faire und genaue Beurteilung der Lehrqualität erlauben. Entsprechend müssen die genutzten Erhebungsinstrumente eine hohe Reliabilität und Validität aufweisen. In dieser Übersichtsarbeit wird die verfügbare Literatur zur Evaluation des Medizinstudiums mit Hinblick auf die möglichen Dimensionen der Lehrqualität, die psychometrischen Eigenschaften der Instrumente und potentielle Störfaktoren dargestellt.

**Methoden:** Ausgehend von einer Schlagwortsuche in Pubmed, PsycINFO und PSYNDEX wurde eine Literatur-Recherche zur Evaluation im Medizinstudium durchgeführt. Berücksichtigung fanden Arbeiten, die bis zum 30.6.2011 in die Datenbanken aufgenommen wurden sowie „graue Literatur“. Die Ergebnisse werden in narrativer Form präsentiert.

**Ergebnisse:** Es wurden vier Dimensionen der Lehrqualität im Medizinstudium identifiziert: Strukturen, Prozesse, Dozenten-Charakteristika und das Lehr-Ergebnis. Zur Betrachtung der ersten drei Dimensionen werden in erster Linie studentische Bewertungen herangezogen. Hierfür liegen einige reliable, in deutscher Sprache verfügbare Instrumente vor. Die Validität studentischer Bewertungen wird jedoch durch zahlreiche potentielle Störfaktoren eingeschränkt. Zur Beurteilung des Lehr-Ergebnisses werden vor allem Prüfungsleistungen herangezogen, deren Nutzbarkeit allerdings aufgrund methodischer Probleme eingeschränkt sein kann. Zudem genügen nicht alle Prüfungen an deutschen medizinischen Fakultäten den gängigen Qualitätsstandards.

**Folgerung:** Die Auswahl von Instrumenten zur Evaluation des Medizinstudiums sollte sich daran orientieren, welche Dimension der Lehre beurteilt werden soll. Entsprechend können Evaluationsergebnisse auch nur vor dem Hintergrund des vom genutzten Erhebungsinstrument abgebildeten Konstrukts und dessen spezifischen Störfaktoren interpretiert werden.

## Introduction

Medical education must meet high standards because medical school graduates – mainly physician practitioners – carry great responsibility. In order to assess the quality of education, evaluations are performed at all German medical schools. No less than 10 years ago, the German Society for Evaluation established standards for the evaluation of university level education. According to these standards, evaluation instruments must permit a fair, accurate, and reliable assessment of teaching quality [1]. Medical education differs from other study programs in that it offers restricted choice of courses and uses unique teaching formats such as problem-based learning and bedside teaching [2], [3]. Seemingly generic teaching formats (e.g., lectures) may be supplemented by elements specific to medical education (e.g., live presentations of patient case histories). Thus, it is questionable whether evaluation instruments from other study programs can readily be transferred to medical education. In general, to assess the reliability and, in particular, the validity of evaluation procedures, the construct of ‘good teaching’ underlying an evaluation instrument must be known. This article presents the results of a broad literature search on ‘evaluation in medical education’, funded by the Association of the Scientific Medical Societies in Germany (Arbeitsgemeinschaft der Wissenschaftlichen Medizinischen Fachgesellschaften e.V., AWMF). Search results were discussed by a joint committee of the AWMF and the Medizinische Fakultätentag (MFT). The literature search intended to answer the following questions:

1. Which dimensions of teaching quality can be assessed in the context of medical education?
2. Which evaluation instruments are currently used, and which outcomes do they target?
3. What are the psychometric properties of these evaluation instruments?
4. What needs to be considered when designing questionnaires for evaluation in medical education, and which confounding factors must be considered when interpreting results?

## Methods

In order to address these questions, we conducted a comprehensive literature search including original research, systematic reviews, dissertations and the so-called ‘grey literature’ published in German or English. We searched Pubmed, PsycINFO, and PSYNDEX (keywords: ‘medical education’, ‘undergraduate medical education’, ‘medical curriculum’ combined with ‘evaluation’, ‘evaluation of teaching effectiveness’ and ‘student ratings’ and their German translations: ‘Medizinische Ausbildung’, ‘Medizinstudium’/‘Studium der Medizin’, ‘medizinisches Curriculum’, ‘Evaluation’, ‘Lehrevaluation’, ‘studentische Bewertungen’) for relevant articles that have been added to the respective databases up to July 30, 2011.

Additional relevant papers were identified from reference lists of published reports. In addition, we searched the online archives of the following journals: Deutsche Medizinische Wochenschrift, GMS Zeitschrift für Medizinische Ausbildung, Hochschulmanagement, Qualität in der Wissenschaft as well as Wissenschaftsmanagement. We consulted experts in the field of medical education for recommendations of relevant articles and used Google to find additional publications. The literature was analysed until saturation was reached (i.e., until no additional content was identified with respect to the research questions).

During a second, more in-depth analysis of identified publications, we extracted those articles that provided answers to the four research questions. Content extraction was guided by a checklist prompting researchers to enter information on the dimension of teaching quality assessed as well as the data collection tool (if available along with its psychometric properties, such as Cronbach’s alpha).

## Results

A total of 116 articles were retrieved. Of these, 46 were found in Pubmed, 22 in PsychINFO, and 4 in PSYNDEX. In addition, 28 articles were identified in the online archives of the above-mentioned German journals. The remaining 16 articles were identified as secondary literature, by recommendation, or via Internet search engines. A complete list of all 116 articles is available in Attachment 1. Many of these articles were not specific to medical education, but focused on general issues related to evaluation of university level teaching. Furthermore, not all articles provided specific answers to the aforementioned research questions. In order to answer the first three research questions, we included 30 articles with a specific focus on medical education. With respect to the fourth research question, hardly any relevant results were identified in the literature specific to medical education. Thus, 14 additional articles without a specific focus on medical education were included. The complete list of all 116 identified articles provides information on which articles were used to answer the research questions.

Due to the broadly defined research questions and, consequently, due to the high structural and content-related heterogeneity of the identified articles, we decided to present the results in form of a narrative. This approach is currently being recommended for review articles that are mainly based on quasi-experimental studies. In this context, numerical analyses (e.g., meta-analyses) seem less well-suited to answer relevant research questions because they unnecessarily constrain the range of contents covered [4]. According to current perspectives in the field of medical didactics [5], if performed according to good scientific practice, narrative reviews may yield higher informational value than averaged figures.

The results section is organised according to the four research questions. For the first three questions, it is further structured according to the four dimensions of teaching, which are specified in the following section.

### Question 1: Dimensions of teaching quality in medical education

All target parameters used to assess teaching quality described in the published literature can be categorised into four dimensions [6]: On the curricular level, structural (first) as well as procedural (second) aspects of teaching can be considered; the third quality criterion refers to teacher characteristics, and the fourth dimension refers to the outcome of teaching activities. The structural dimension comprises, for instance, the physical environment available for teaching, teaching materials as well as the design of a curriculum. The learning process refers to aspects such as teacher-student interaction or teaching/learning atmosphere. Instructor-specific characteristics include teaching skills and the level of preparation, but also the teachers’ enthusiasm as perceived by their students. The outcome dimension describes aspects such as learning outcome and the development of professional attitudes as a result of teaching.

Structures and processes related to teaching are assessed by many of the published evaluation instruments (see Question 2), especially because data collection and analysis can be automated easily. A reliable and valid assessment of individual teacher performance is a more complex endeavour. The corresponding instruments must meet high psychometric standards, especially due to potential consequences of such evaluation results for individual careers.

Defining teaching quality by related outcomes appears straightforward. In this context, Blumberg [7] suggests three types of outcomes: She defines ‘educational outcome’ as the development of competencies for independent, life-long learning. The term ‘clinical career outcomes’ comprises competencies relevant for the medical profession (see also [8]). Blumberg defines ‘environmental outcomes’ as the development of professional attitudes towards teaching itself – in the sense that graduates consider passing on their knowledge and skills to their younger colleagues as part of their own professional role, thus shaping the environment at their teaching institutions. To date, there is no consensus on how to operationalise these three types of educational outcomes.

### Question 2: Target outcomes and assessment instruments

As mentioned above, the following description of target outcomes and assessment instruments is guided by the four dimensions of teaching quality (structure, process, teacher, and outcome). Given that a clear-cut alignment between dimensions and individual instruments (and vice versa) is not always possible, we will elaborate on the available instruments in the context of the dimension primarily targeted. A summary of all identified instruments with a focus on medical education is presented in Table 1 (Tab. 1).

Educational *structures and processes* are mainly evaluated using self-administered questionnaires that are completed by students. Some of the available instruments cover both structures and processes (“Medical Student Experience Questionnaire”; MedSEQ; 32 items [9] and “Marburger Fragebogen zur Evaluation des Lehrangebots in der Medizin”; 12 items [10]). Four additional instruments focus mainly on teaching-related processes and, in this context, use the term ‘learning environment’. The “Dundee Ready Education Environment Measure” (DREEM; 50 items [11]) has recently become available in German [12]. The comprehensive “Learning Environment Questionnaire” (LEQ; 65 items [13]) yields some overlap with the more concise “Measuring the School Learning Environment Survey” (MSLES; 50 items [14]).

The “Medical Instructional Quality” (MedIQ; 25 items [15]) was specifically designed for evaluating clinical teaching. It covers four aspects of clinical teaching related to outpatient settings. Among other factors, the MedIQ focuses on the clinical learning environment as well as the participation of students in patient care. A comprehensive review of additional instruments for evaluating the learning environment was published in 2010 [16].

Numerous instruments have been designed to evaluate *individual teachers* (see Table 1 (Tab. 1)). Again, self-administered questionnaires are predominantly used, in most cases containing scaled items and open answer options. Most instruments specific to medical education and assessing individual teacher performance are tailored to the clinical teaching context (e.g. bedside teaching) rather than lectures and seminars. Detailed information on the available instruments can be found in Table 1 (Tab. 1). There is one noteworthy questionnaire for the assessment of teaching in outpatient settings (“Student Evaluation of Teaching in Outpatient Clinics”; SETOC [17]). Furthermore, the SFDP-26 (“Stanford Faculty Development Program” [18]) survey, which is also available in German [19] needs to be mentioned in this context. This tool was originally developed at the Mayo Clinic and, by mapping the seven “Stanford-Criteria for Good Teaching”, is well-grounded in theory.

As described above, the *outcome of teaching*, i.e. student learning outcome, is reflected not only in the accumulation of knowledge and practical skills but also in the development of professional attitudes [7], [8]. Unfortunately, we did not find any instruments covering the full range of these outcomes. Some German medical schools use student performance in the written part of the second state examination as a surrogate parameter for teaching quality [20]. However, multiple-choice (MC) questions (such as those used in state examinations) mainly assess factual knowledge. By memorising the correct answer [21] or by deliberate practice of MC-questions [22], students may improve their exam results regardless of their actual knowledge. Similar limitations pertain to the Progress Test, which is used by some German medical schools. This formative assessment, which is applied repeatedly during the course of the curriculum, also uses MC-questions. Nevertheless, it is considered a useful and important source of information for students as well as curriculum evaluation owing to its longitudinal and cross-sectional design [23].

In general, state examinations are characterised by high internal consistency. However, learning outcomes of individual classes/courses of a given curriculum can only be assessed by analysing the exam results that were performed at the medical schools. According to a recent analysis, these exams often do not meet current quality standards [24]. Recently, an evaluation tool estimating student learning outcome from comparative self-assessments has been developed as an alternative. The tool’s main advantage over end-of-course exams is its adjustment for initial student performance levels, thus facilitating a critical appraisal of the learning outcome created during a course [25].

Finally, surveys among medical school graduates can be used to assess the quality of medical education. In principle, all four dimensions of teaching quality may be measured with this method. However, the present literature search identified neither articles specific to medical education nor studies related to other types of university level teaching that systematically evaluated the quality of instruments used for this purpose.

### Question 3: Psychometric properties of assessment instruments

Questionnaires as well as exam results may be analysed regarding their reliability and validity. The *reliabilities* of the instruments used to assess structural and procedural aspects of teaching are given in the last column of Table 1 (Tab. 1). Cronbach’s α, signifying the lower limit of reliability, is satisfactory for most questionnaires. Interrater reliability of evaluation data depends on the numbers of completed questionnaires [26]. However, no studies have yet reported a minimum response rate that would be necessary for results to be deemed reliable (see below). Measuring the reliability of examinations is a prerequisite for using exam results for evaluation purposes. At German medical schools, however, these analyses are performed on less than 40% of summative exams [24].

A well-founded interpretation of evaluation results requires the data to be valid. While content validity of examinations and evaluation instruments is usually acceptable, data on criterion and construct validity is often lacking. In addition, confounding factors potentially impacting the validity of results need to be considered. Such factors have mainly been identified for *student ratings*, and they are being discussed below (Question 4). However, the considerations pertaining to this aspect are mainly based on literature with no direct link to medical education.

The validity of *examinations* is threatened mainly by two confounding factors [27]. Construct under-representation exists if the construct to be evaluated by the exam is not completely covered. In this case, students have an advantage if they accidentally focus their learning on those contents that are covered by the exam. The second essential confounding factor is construct-irrelevant variance. This occurs if, for instance, exam questions are constructed sub-optimally, so that the exam assesses not only obvious content knowledge but also students’ abilities to cope with questions that are difficult to understand. Due to a lack of valid external criteria and necessary resources, criterion validity of examinations is usually not evaluated. The above-mentioned instrument for calculating student learning outcomes from comparative self-assessments has been shown to be construct-valid in a first study [25]. Additional published results were not available at the time of the literature search. Similarly, we did not identify any studies on the reliability and validity of graduate surveys.

### Question 4: Questionnaire design and confounding factors 

The most common evaluation instrument in practice as well as in the identified publications is the self-administered questionnaire. When designing and using questionnaires, several aspects must be considered. As mentioned above, hardly any articles addressing this question were identified. Thus, below we present some of the pertinent findings related to questionnaire design and the most important confounding factors of self-administered evaluation instruments, mainly without a direct link to medical education. 

Question type, scale options and data collection procedures may all impact on the psychometric properties of questionnaires. With respect to question type, there are open questions and scaled items. Free-text comments can yield valuable qualitative information, but not every student volunteers their opinion. Scaled items lend themselves to quantitative analyses. Global ratings that are frequently used to obtain an overall appraisal of a course (e.g., using school grades) are criticized by some authors due to their susceptibility to confounding (see below) [28], [29]. Other authors contend that the construct of good teaching is virtually one-dimensional and thus can well be assessed using global ratings [30]. Additional studies show that the reliability of instruments is positively related to the number of specific items contained [31], [32].

Scaled questions yield more favourable ratings if the positive anchor is placed on the left [33]. Furthermore, the wording of items may be interpreted differently by individual students [3]. In addition, the evaluation procedure itself needs to be considered. This factor becomes increasingly important because many medical schools have moved their evaluations to online platforms. In general, online evaluations yield lower response rates than traditional paper-based evaluations. While one study did not demonstrate an effect of this on evaluation results (in fact, students provided even more comments on the online version) [34], another report stated that low-performing students were less likely to participate in online evaluations than their high-performing peers [35]. In addition, anonymous evaluations typically yield less favourable ratings than evaluations requiring students to provide identifying information [36]. With respect to graduate surveys, it should be considered that evaluation results tend to get worse the more time has passed between exposure to teaching and data collection [37].

Items that are used to evaluate individual teachers are particularly prone to confounding. It has been shown that teachers who are enthusiastic and who have a good reputation systematically receive more favourable ratings [38], even if the content they present is flawed [39], [40]. Another important confounding factor is student interest in a course [41], [42]: Courses with voluntary participation typically receive more positive ratings than compulsory courses [28], [43]. Moreover, well-attended courses are generally evaluated more positively [44]. In the context of medical education, teaching in subjects related to basic science and theoretical medicine tend to receive less favourable ratings than clinical teaching. Similarly, lectures yield worse evaluations than small-group formats [37].

## Discussion

The present article is a broad review of the available literature on evaluation in medical education. The results suggest that teaching quality is not a univariate construct. Rather, all four – partially overlapping – dimensions (‘structure’, ‘process’, ‘instructor’, and ‘outcome’) can and should be considered in evaluations. In addition, interpretation of evaluation results needs to be informed by the construct underlying the data collection tool. For instance, student appraisals of a teacher’s punctuality or the condition of classrooms do not allow direct conclusions to be drawn on student learning outcomes. Exam results may be used to estimate learning outcome. However, they merely reflect performance at one point in time and do not provide information on progress during a course. Progress testing is one solution to this problem, but given that it is solely based on multiple choice questions it is unable to assess practical skills or professional attitudes. In addition, it does not use a pre-post design, which would be necessary to evaluate individual courses or modules (as opposed to student cohorts or entire study programs).

The quantitative analysis of evaluation data (e.g., by calculating means of global course ratings provided by students using a grading system) facilitates comparisons across courses. However, this approach entails two risks: First, global ratings are unlikely to represent a clear-cut construct. Second, such ratings are prone to several confounding factors [45]. If one assumes that teaching quality with all its facets can be reflected by one single mean rating, both risks threaten the reliability and validity of such global assessments. In addition to the confounding factors mentioned above, the length of data collection tools should be mentioned at this point. Some of the questionnaires listed in Table 1 (Tab. 1) contain more than 60 items and are probably not well-suited for frequent and regular use in course evaluations due to low student acceptance [46].

Less than half of the articles identified in the initial search were included in this review. The main reason for exclusion was a lack of relatedness to medical education. For instance, the validated questionnaire SEEQ (“Students’ Evaluation of Educational Quality”) [47] is widely used in higher education institutions in the United States. It is unclear to which extent this instrument can be generalized to medical education as its items are not specific for medical education. In addition, this questionnaire was developed for higher education in the U.S. which differs from the German setting in some respect. German instruments used to evaluate (non-medical) teaching are the HILVE (“Heidelberger Inventar zur Lehrveranstaltungs-Evaluation”) [48] and the HILVE II. Both tools possess good psychometric properties, but again generalisability to medical education is questionable. Due to the specifics of medical education mentioned above, further psychometric testing is definitely advisable before applying this tool. 

The results of this literature review do not justify general recommendations to be made for the use of specific questionnaires to evaluate medical education in Germany. One reason for this is that the choice of the data collection tool should be guided by the goal of evaluation. However, a preliminary and resource efficient solution could be to use the Marburger questionnaire (for structural and procedural aspects) and the SFDP-26 German [19] (for teachers), as they are already available in German and possess good psychometric characteristics. Since those instruments that were mainly developed and validated in English-speaking countries cannot easily be transferred to the context of medical education in Germany, a medium-term goal should be to design a new questionnaire from existing and new items and validate this new tool in German medical schools. This process should be informed by psychometric expertise and could involve several German medical schools as part of a related research project. By using an instrument that has been mutually agreed upon at multiple locations, greater comparability of the results could be achieved. A possible development and implementation strategy is currently being discussed between MFT and AWMF. 

There is a risk that relevant publications have not been included in our final selection of papers for this review. The main limitation of the present article is that the majority of included studies were done in English-speaking countries where medical education can differ substantially from Germany (e.g., clerkships cannot readily be compared to the German ‘Blockpraktikum’ and ‘Famulatur’; there is no direct equivalent to the ‘Praktische Jahr’ in most English-speaking countries). In addition, the sources used for answering the fourth research question were largely not specific to medical education. At best, it is questionable if the insights into questionnaire design and confounding factors as they pertain to evaluation in other disciplines can readily be transferred to medical education. Finally, our search for published instruments used to assess teaching quality mainly identified self-administered questionnaires that are completed by students. Other data collection procedures (e.g., graduate surveys) might also provide helpful information. Due to limited data, we chose not to discuss these instruments in the present review.

## Conclusion

The evaluation of medical education is mainly based on student ratings of structural and procedural aspects of teaching as well as the performance of individual teachers. The present review identified several reliable instruments to assess these three dimensions of teaching quality. However, evaluation research unrelated to medicine has identified a number of confounding factors impacting on student ratings, thereby threatening the validity of these instruments. These confounding factors should be considered or re-addressed when using student ratings to evaluate medical education. In Germany, the assessment of teaching quality based on exam performance is problematic as there is currently no comprehensive quality control of summative exams at German medical schools. Graduate surveys are not widely used and rely on instruments with unknown validity and reliability.

## Clinical and practical implications

- The quality of medical education is a multi-dimensional construct; the four basic dimensions for assessing teaching quality are structures, processes, teacher characteristics, and learning outcome.
- To assess structures, processes and individual teachers in medical education, several instruments with good psychometric characteristics are available. The assessment of learning outcome is limited mainly due to unknown or insufficient reliability and validity of summative exams in medical schools.
- When designing and implementing evaluation instruments, the confounding factors presented in this review must be taken into account as far as they are likely to generalise from other fields of university level teaching to medical education.

## Notes

### Competing interests

The authors declare that they have no competing interests.

### Authorship

The authors Herrmann-Lingen C and Raupach T contributed equally to this work.

## Supplementary Material

Complete list of the literature

## Figures and Tables

**Table 1:**
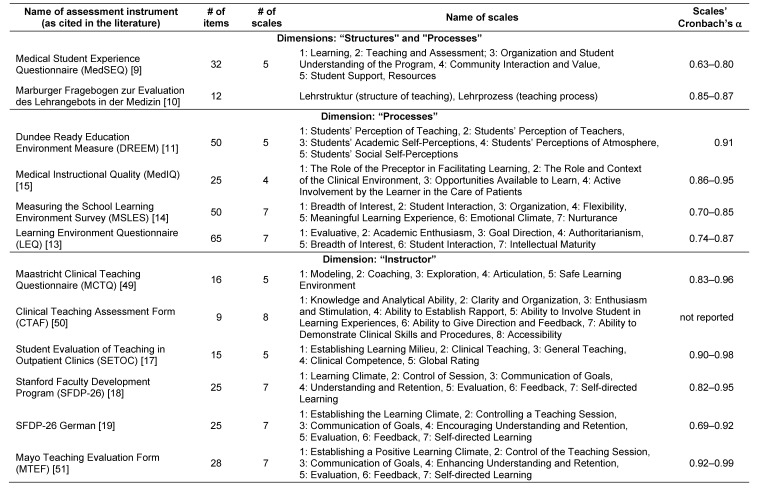
Summary of all identified evaluation instruments for teaching quality
