# Supplementary material for: Evaluation in medical education: A topical review of target parameters, data collection tools and confounding factors
Source: Ger Med Sci. 2015 Sep 16;13:Doc15. doi: 10.3205/000219 (PMC4576315; doi:10.3205/000219)
Supplement: Complete list of the literature [file GMS-13-15-s-001.pdf]

Appendix to: Schiekirka S, Feufel MA, Herrmann-Lingen C, Raupach T. Evaluation in medical education: A topical review of target parameters, data collection tools and confounding factors.
GMS Ger Med Sci. 2015;13:Doc15. DOI: 10.3205/000219

## Complete list of the literature – Aufstellung der Gesamtliteratur


1. **Abrami PC, D'Apollonia S, Cohen PA. Validity of student ratings of instruction: What we know and what we do not. J Educ Psychol. 1990;82(2):219-31. DOI: 10.1037/0022-0663.82.2.219
2. **Adams MJ, Umbach PD. Nonresponse and online student evaluations of teaching: Understanding the influence of salience, fatigue, and academic environments. Res High Educ. 2012;53(5):576-91. DOI: 10.1007/s11162-011-9240-5
3. *Afonso NM, Cardozo LJ, Mascarenhas OA, Aranha AN, Shah C. Are anonymous evaluations a better assessment of faculty teaching performance? A comparative analysis of open and anonymous evaluation processes. Fam Med. 2005 Jan;37(1):43-7.
4. *Albanese M, Prucha C, Barnet JH, Gjerde CL. The effect of right or left placement of the positive response on Likert-type scales used by medical students for rating instruction. Acad Med. 1997 Jul;72(7):627-30. DOI: 10.1097/00001888-199707000-00015
5. **Aleamoni LM. Student rating myths versus research facts from 1924 to 1998. J Pers Eval Educ. 1999;13(2):153-66. DOI: 10.1023/A:1008168421283
6. Anderson J. Multiple choice questions revisited. Med Teach. 2004 Mar;26(2):110-3. DOI: 10.1080/0142159042000196141
7. Arnold-Fabian U. Vergleich der Leistungsorientierten Mittelverteilung (LOM) an deutschen Medizinischen Fakultäten – eine Bestandsaufnahme [Dissertation]. Mainz: Johannes Gutenberg-Universität; 2010.
8. Ballantyne C. Online evaluations of teaching: An examination of current practice and considerations for the future. New Dir Teach Learn. 2003;2003(96):103-12. DOI: 10.1002/tl.127
9. Balsmeier B, Lück S. Qualität von Modulen in Lehre und Studium. Qualität in der Wissenschaft. 2008;(2):49-53.
10. Baroffio A, Nendaz MR, Perrier A, Vu NV. Tutor training, evaluation criteria and teaching environment influence students' ratings of tutor feedback in problem-based learning. Adv Health Sci Educ Theory Pract. 2007 Nov;12(4):427-39. DOI: 10.1007/s10459-006-9008-4
11. Beckman TJ, Ghosh AK, Cook DA, Erwin PJ, Mandrekar JN. How reliable are assessments of clinical teaching? A review of the published instruments. J Gen Intern Med. 2004 Sep;19(9):971-7. DOI: 10.1111/j.1525-1497.2004.40066.x
12. Beckman TJ, Lee MC, Mandrekar JN. A comparison of clinical teaching evaluations by resident and peer physicians. Med Teach. 2004 Jun;26(4):321-5. DOI: 10.1080/01421590410001678984
13. *Beckman TJ, Lee MC, Rohren CH, Pankratz VS. Evaluating an instrument for the peer review of inpatient teaching. Med Teach. 2003 Mar;25(2):131-5. DOI: 10.1080/0142159031000092508
14. Berger U, Buhl T. Praktische Durchführung einer Lehrveranstaltungsevaluation: Qualität - Arbeitsschritte - Kosten - Konsequenzen. Med Ausbild. 2000;17(2):110-5. Available from: https://gesellschaft-medizinische-ausbildung.org/files/ZMA-Archiv/2000/2/Berger_U,_Buhl_T.pdf
15. Berger U, Schleußner C. Hängen Ergebnisse einer Lehrveranstaltungs-Evaluation von der Häufigkeit des Veranstaltungsbesuches ab? Z Päd Psychol. 2003;17(2):125–31. DOI: 10.1024//1010-0652.17.2.125
16. Berger U, Schleussner C, Strauss B. Umfassende Lehrevaluation in der Medizin – eine Aufgabe für die psychosozialen Fächer [Comprehensive evaluation of medical teaching – a task for the psychosocial disciplines?]. Psychother Psychosom Med Psychol. 2003 Feb;53(2):71-8. DOI: 10.1055/s-2003-36966


*=specific to medical education and cited in the article / medizinspezifisch und in dem Artikel zitiert
**=not specific to medical education and cited in the article / nicht-medizinspezifisch und in dem Artikel zitiert

17. *Billings-Gagliardi S, Barrett SV, Mazor KM. Interpreting course evaluation results: insights from thinkaloud interviews with medical students. Med Educ. 2004 Oct;38(10):1061-70. DOI: 10.1111/j.1365-2929.2004.01953.x
18. *Blumberg P. Multidimensional outcome considerations in assessing the efficacy of medical educational programs. Teach Learn Med. 2003;15(3):210-4. DOI: 10.1207/S15328015TLM1503_10
19. *Boyle P, Grimm MC, McNeil HP, Scicluna H. The UNSW Medicine Student Experience Questionnaire (MedSEQ). San Francisco: Academia; 2009. Available from: http://www.academia.edu/5252480/Medicine_Student_Experience_Questionnaire_MEDSEQ_UNSW
20. Braun E, Leidner B. Academic course evaluation: Theoretical and empirical distinctions between self-rated gain in competences and satisfaction with teaching behavior. Eur Psychol. 2009;14(4):297-306. DOI: 10.1027/1016-9040.14.4.297
21. Cantillon P. Evaluation: beyond the rhetoric. J Eval Clin Pract. 1999 Aug;5(3):265-8. DOI: 10.1046/j.1365-2753.1999.00175.x
22. **Cashin WE. Student ratings of teaching: A summary of the research. East Lansing, MI, USA: Office of Faculty and Organizational Development at Michigan State University; 1988. (IDEA Paper; No.20). Available from: http://ideaedu.org/wp-content/uploads/2014/11/idea-paper_50.pdf
23. Chenot JF, Ehrhardt M. Objective structured clinical examination (OSCE) in der medizinischen Ausbildung: Eine Alternative zur Klausur. Z Allg Med. 2003;79:437–42. DOI: 10.1055/s-2003-43064
24. Dagenais ME, Hawley D, Lund JP. Assessing the effectiveness of a new curriculum: Part I. J Dent Educ. 2003 Jan;67(1):47-54.
25. **d'Apollonia S, Abrami PC. Navigating student ratings of instruction. Am Psychol. 1997;52(11):1198-208. DOI: 10.1037/0003-066X.52.11.1198
26. *Deutsche Gesellschaft für Evaluation e.V., editor. Standards für Evaluation. Köln: DeGEval; 2002.
27. *Downing SM, Haladyna TM. Validity threats: overcoming interference with proposed interpretations of assessment data. Med Educ. 2004;38(3):327-33. DOI: 10.1046/j.1365-2923.2004.01777.x
28. Elzubeir M, Rizk D. Evaluating the quality of teaching in medical education: are we using the evidence for both formative and summative purposes? Med Teach. 2002 May;24(3):313-9. DOI: 10.1080/01421590220134169
29. *Frank JR, Danoff D. The CanMEDS initiative: implementing an outcomes-based framework of physician competencies. Med Teach. 2007 Sep;29(7):642-7. DOI: 10.1080/01421590701746983
30. *Freeman A, Van Der Vleuten C, Nouns Z, Ricketts C. Progress testing internationally. Med Teach. 2010;32(6):451-5. DOI: 10.3109/0142159X.2010.485231
31. Galbraith CS, Merrill GB, Kline DM. Are student evaluations of teaching effectiveness valid for measuring student learning outcomes in business related classes? A neural network and Bayesian analyses. Res High Educ. 2012;53(3):353-74. DOI: 10.1007/s11162-011-9229-0
32. Germain ML, Scandura TA. Grade inflation and student individual differences as systematic bias in faculty evaluations. J Instruct Psychol. 2005;32(1):58-67.
33. Gesellschaft für Medizinische Ausbildung; Kompetenzzentrum Prüfungen Baden-Württemberg; Fischer MR. Leitlinie für Fakultäts-interne Leistungsnachweise während des Medizinstudiums: Ein Positionspapier des GMA-Ausschusses Prüfungen und des Kompetenzzentrums Prüfungen Baden-Württemberg. GMS Z Med Ausbild. 2008;25(1):Doc74. Available from: http://www.egms.de/en/journals/zma/2008-25/zma000558.shtml

*=specific to medical education and cited in the article / medizinspezifisch und in dem Artikel zitiert
**=not specific to medical education and cited in the article / nicht-medizinspezifisch und in dem Artikel zitiert

34. *Gibson KA, Boyle P, Black DA, Cunningham M, Grimm MC, McNeil HP. Enhancing evaluation in an undergraduate medical education program. Acad Med. 2008 Aug;83(8):787-93. DOI: 10.1097/ACM.0b013e31817eb8ab
35. Giesler M, Forster J, Biller S, Fabry G. Entwicklung eines Fragebogens zur Erfassung von Kompetenzen in der Medizin: Ergebnisse zur Reliabilität und Validität [Development of a questionnaire to assess medical competencies: Reliability and validity of the Questionnaire]. GMS Z Med Ausbild. 2011;28(2):Doc31. DOI: 10.3205/zma000743
36. **Griffin BW. Instructor reputation and student ratings of instruction. Contemp Educ Psychol. 2001 Oct;26(4):534-52. DOI: 10.1006/ceps.2000.1075
37. Haladyna TM, Downing SM. Construct-Irrelevant Variance in High-Stakes Testing. Educ Meas. 2004;23(1):17-27. DOI: 10.1111/j.1745-3992.2004.tb00149.x
38. Hamdy H, Prasad K, Anderson MB, Scherpbier A, Williams R, Zwierstra R, Cuddihy H. BEME systematic review: predictive values of measurements obtained in medical schools and future performance in medical practice. Med Teach. 2006 Mar;28(2):103-16. DOI: 10.1080/01421590600622723
39. Harden RM. Learning outcomes as a tool to assess progression. Med Teach. 2007 Sep;29(7):678-82. DOI: 10.1080/01421590701729955
40. *Herzig S, Marschall B, Nast-Kolb D, Soboll S, Rump LC, Hilgers RD. Positionspapier der nordrhein-westfälischen Studiendekane zur hochschulvergleichenden leistungsorientierten Mittelvergabe für die Lehre [Distribution of government funds according to teaching performance]. GMS Z Med Ausbild. 2007;24(2):Doc109. Available from: http://www.egms.de/en/journals/zma/2007-24/zma000403.shtml
41. Himmelbauer M. Summative Integrierte Multiple Choice-Prüfungen: Ein Promotor oder eine Hindernis für bedeutungsorientiertes Lernen der Studierenden [Summative integrative multiple choice examinations: Do they promote or prevent deep learning approaches?]. GMS Z Med Ausbild. 2009;26(4):Doc42. DOI: 10.3205/zma000635
42. Hobson SM, Talbot DM. Understanding student evaluations: what all faculty should know. Coll Teach. 2001;49(1):26-31. DOI: 10.1080/87567550109595842
43. *Iblher P, Zupanic M, Härtel C, Heinze H, Schmucker P, Fischer MR. Der Fragebogen "SFDP26-German": Ein verlässliches Instrument zur Evaluation des klinischen Unterrichts [The Questionnaire "SFDP26-German": a reliable tool for evaluation of clinical teaching]? GMS Z Med Ausbild. 2011;28(2):Doc30. DOI: 10.3205/zma000742
44. *Irby DM, Gillmore GM, Ramsey PG. Factors affecting ratings of clinical teachers by medical students and residents. Acad Med. 1987;62(1):1-7. DOI: 10.1097/00001888-198701000-00001
45. **Jackson DL, Teal CR, Raines SJ, Nansel TR, Force RC, Burdsal CA. The dimensions of students' perceptions of teaching effectiveness. Educ Psychol Meas. 1999;59(4):580-96. DOI: 10.1177/00131649921970035
46. Jaeger M, Dannenberg M, Wülbern K. Formelgebundene Mittelvergabe innerhalb von Fakultäten: Ausgestaltungstrends und Umsetzungsbeispiele. Hochschulmanagement. 2007;2(1):9-14.
47. *James PA, Osborne JW. A measure of medical instructional quality in ambulatory settings: the MedIQ. Fam Med. 1999 Apr;31(4):263-9.
48. Johnson, T. Online student ratings: will students respond? Paper presented at the AERA. Annual conference of the American Educational Research Association; 2002; New Orleans, LA.

*=specific to medical education and cited in the article / medizinspezifisch und in dem Artikel zitiert
**=not specific to medical education and cited in the article / nicht-medizinspezifisch und in dem Artikel zitiert

49. Jones RF, Froom JD. Faculty and administration views of problems in faculty evaluation. Acad Med. 1994 Jun;69(6):476-83. DOI: 10.1097/00001888-199406000-00015
50. Kern DE, Thomas PA, Howard DM, Bass EB. Curriculum development for medical education – A six-step approach. Baltimore and London: The John Hopkins University Press; 1998.
51. *Kogan JR, Shea JA. Course evaluation in medical education. Teach Teach Educ. 2007;23(3):251-64. DOI: 10.1016/j.tate.2006.12.020
52. Kohler N, van den Bussche H. Je schwieriger, desto beliebter. Nutzen, didaktische Qualität und Schwierigkeitsgrad des vorklinischen Lehrangebots aus der Sicht von Hamburger Medizinstudenten. Ann Anat. 2004;186(3):283-8. DOI: 10.1016/S0940-9602(04)80018-9
53. *Krebs K. Marburger Fragebogen zur Evaluation des Lehrangebots in der Medizin: Eine Untersuchung zur Reliabilität und Dimensionalität des Marburger Fragebogens zur Evaluation des Lehrangebots am Fachbereich Medizin [Dissertation]. Marburg: Philipps-Universität Marburg; 2006. Available from: http://archiv.ub.uni-marburg.de/diss/z2006/0387/pdf/dkk.pdf
54. Krempkow R. Leistungsbewertung, Leistungsanreize und die Qualität der Hochschullehre. Qualität in der Wissenschaft. 2007;1(2):38-43.
55. Krempkow R. Von Zielen und Indikatoren – Versuch einer Operationalisierung für Lehre und Studium im Rahmen eines Quality Audit. Qualität in der Wissenschaft. 2009;3(1+2):44-53.
56. Krempkow R, Vissering A, Wilke U, Bischof L. Absolventenstudien als „outcome evaluation“. Sozialwissenschaften und Berufspraxis (SuB). 2010;33(1):43-63. Available from: http://www.wissenschaftsmanagement-online.de/sites/www.wissenschaftsmanagement-online.de/files/migrated_wimoarticle/Krempkow_u.a._2010_Absolventenstudienalsoutcomeevaluation.pdf
57. Kubinger KD, Holocher-Ertl S, Reif M, Hohensinn C, Frebort M. On Minimizing Guessing Effects on Multiple-Choice Items: Superiority of a two solutions and three distractors item format to a one solution and five distractors item format. Int J Select Assess. 2010;18(1):111-5. DOI: 10.1111/j.1468-2389.2010.00493.x
58. Kulik JA. Student ratings: validity, utility, and controversy. New Dir Instit Res. 2001;2001(109):9-25. DOI: 10.1002/ir.1
59. *Litzelman DK, Stratos GA, Marriott DJ, Skeff KM. Factorial validation of a widely disseminated educational framework for evaluating clinical teachers. Acad Med. 1998;73(6):688-95. DOI: 10.1097/00001888-199806000-00016
60. Lloyd-Jones G, Fowell S, Bligh JG. The use of the nominal group technique as an evaluative tool in medical undergraduate education. Med Educ. 1999;33(1):8-13. DOI: 10.1046/j.1365-2923.1999.00288.x
61. Lück S. Studentische Lehrevaluationen im Rahmen neuer Studienstrukturen. Hochschulmanagement. 2007;2:40-6.
62. *Mahamed A, Gregory PA, Austin Z. "Testwiseness" among international pharmacy graduates and Canadian senior pharmacy students. Am J Pharm Educ. 2006 Dec;70(6):131. DOI: 10.5688/aj7006131
63. Mallich K, Kirnbauer I, Schmidts M, Stowasser-Bloch K. Konzept zum Qualitätsmanagement in Studium und Lehre an der Medizinischen Universität in Wien mittels Abbildung von Prozesslandschaften und Definition von Leistungskriterien. Qualität in der Wissenschaft. 2007:1(3):72-6.

*=specific to medical education and cited in the article / medizinspezifisch und in dem Artikel zitiert
**=not specific to medical education and cited in the article / nicht-medizinspezifisch und in dem Artikel zitiert

64. Mallon WT, Jones RF. How do medical schools use measurement systems to track faculty activity and productivity in teaching? Acad Med. 2002 Feb;77(2):115-23. DOI: 10.1097/00001888-200202000-00005
65. Marsh HW. The Influence of Student, Course, and Instructor Characteristics in Evaluations of University Teaching. Am Educ Res J. 1980;17(2):219-37. DOI: 10.3102/00028312017002219
66. **Marsh HW. SEEQ: A reliable, valid, and useful instrument for collecting students' evaluations of university teaching. Brit J Psychol. 1982;52(1):77-95. DOI: 10.1111/j.2044-8279.1982.tb02505.x
67. **Marsh HW. Students' evaluations of university teaching: Dimensionality, reliability, validity, potential baises, and utility. J Educ Psychol. 1984;76(5):707-54. DOI: 10.1037/0022-0663.76.5.707
68. **Marsh HW, Roche LA. Making students' evaluations of teaching effectiveness effective: The critical issues of validity, bias, and utility. Am Psychol. 1997;52(11):1187-97. DOI: 10.1037/0003-066X.52.11.1187
69. **Marsh HW, Ware JE. Effects of expressiveness, content coverage, and incentive on multidimensional student rating scales: New interpretations of the Dr. Fox effect. J Educ Psychol. 1982;74(1):126-34. DOI: 10.1037/0022-0663.74.1.126
70. *Marshall RE. Measuring the medical school learning environment. Acad Med. 1978;53(2):98-104. DOI: 10.1097/00001888-197802000-00003
71. McManus IC, Richards P, Winder BC, Sproston KA. Clinical experience, performance in final examinations, and learning style in medical students: prospective study. BMJ. 1998 Jan;316(7128):345-50. DOI: 10.1136/bmj.316.7128.345
72. McOwen KS, Bellini LM, Morrison G, Shea JA. The Development and Implementation of a Health-System-Wide Evaluation System for Education Activities: Build It and They Will Come. Acad Med. 2009;84(10):1352-9. DOI: 10.1097/ACM.0b013e3181b6c996
73. McOwen KS, Kogan JR, Shea JA. Elapsed time between teaching and evaluation: does it matter? Acad Med. 2008 Oct;83(10):S29-32. DOI: 10.1097/ACM.0b013e318183e37c
74. *Möltner A, Duelli R, Resch F, Schultz JH, Jünger J. Fakultätsinterne Prüfungen an den deutschen medizinischen Fakultäten [School-specific assessment in German medical schools]. GMS Z Med Ausbild. 2010;27(3):Doc44. DOI: 10.3205/zma000681
75. Müller-Hilke B. "Ruhm und Ehre" oder LOM für Lehre? – eine qualitative Analyse von Anreizverfahren für gute Lehre an Medizinischen Fakultäten in Deutschland [A qualitative study to investigate the acceptance of performance-based allocation of resources for the improvement of education at German medical schools]. GMS Z Med Ausbild. 2010;27(3):Doc43. DOI: 10.3205/zma000680
76. *Naftulin DH, Ware JE, Donnelly FA. The Doctor Fox Lecture: a paradigm of educational seduction. J Med Educ. 1973 Jul;48(7):630-5. DOI: 10.1097/00001888-197307000-00003
77. Nouns ZM, Georg W. Progress testing in German speaking countries. Med Teach. 2010;32(6):467-70. DOI: 10.3109/0142159X.2010.485656
78. Oppitz M, Schriek G, Busch C, Shiozawa T, Drews U. Offene Fragen vs. Multiple-Choice-Fragen im 1. Abschnitt des Medizinstudiums: Untersuchung am Beispiel von Topographischer Anatomie [Open-answer questions vs. multiple-choice questions for undergraduate medical exams: a case study in topographical anatomy]. GMS Z Med Ausbild. 2007;24(3):Doc150. Available from: http://www.egms.de/en/journals/zma/2007-24/zma000444.shtml
79. Papinczak T. An exploration of perceptions of tutor evaluation in problem-based learning tutorials. Med Educ. 2010 Sep;44(9):892-9. DOI: 10.1111/j.1365-2923.2010.03749.x

*=specific to medical education and cited in the article / medizinspezifisch und in dem Artikel zitiert
**=not specific to medical education and cited in the article / nicht-medizinspezifisch und in dem Artikel zitiert

80. Peitzman SJ, Nieman LZ, Gracely EJ. Comparison of "fact-recall" with "higher-order" questions in multiple-choice examinations as predictors of clinical performance of medical students. Acad Med. 1990;65(9 Suppl):S59-60. DOI: 10.1097/00001888-199009000-00044
81. **Prave RS, Baril GL. Instructor ratings: Controlling for bias from Initial student interest. J Educ Bus. 1993;68(6):362-6. DOI: 10.1080/08832323.1993.10117644
82. Probert CS, Cahill DJ, McCann GL, Ben-Shlomo Y. Traditional finals and OSCEs in predicting consultant and self-reported clinical skills of PRHOs: a pilot study. Med Educ. 2003 Jul;37(7):597-602. DOI: 10.1046/j.1365-2923.2003.01557.x
83. Putz R. LOM für Lehre. In: Tagungsbericht des ordentlichen Medizinischen Fakultätentages; 22.-23. Mai 2008; Heidelberg. 2008. Available from: http://www.mft-online.de/files/referat_putz.pdf
84. *Raupach T, Münscher C, Beissbarth T, Burckhardt G, Pukrop T. Towards outcome-based programme evaluation: using student comparative self-assessments to determine teaching effectiveness. Med Teach. 2011;33(8):e446-53. DOI: 10.3109/0142159X.2011.586751
85. Reichmann G. Überprüfung der Akzeptanz einer zentralen Evaluation von Lehrveranstaltungen am Beispiel der studentischen Lehrveranstaltungsbewertung an der Universität Graz. Qualität in der Wissenschaft. 2007;1(3):77-81.
86. Reimer M. Wie können Absolventenstudien zum Qualitätsmanagement der Hochschulen beitragen? Erfahrungen des Bayerischen Absolventenpanels. Qualität in der Wissenschaft. 2008;2(3):68-73.
87. Ricketts C, Freeman AC, Coombes LR. Standard setting for progress tests: combining external and internal standards. Med Educ. 2009 Jun;43(6):589-93. DOI: 10.1111/j.1365-2923.2009.03372.x
88. **Rindermann H, Schofield N. Generalizability of Multidimensional Student Ratings of University Instruction Across Courses and Teachers. Res High Educ. 2001;42(4):377-99. DOI: 10.1023/A:1011050724796
89. *Roff S. The Dundee Ready Educational Environment Measure (DREEM)– a generic instrument for measuring students' perceptions of undergraduate health professions curricula. Med Teach. 2005 Jun;27(4):322-5. DOI: 10.1080/01421590500151054
90. *Rothman AI, Ayoade F. The development of a learning environment: a questionnaire for use in curriculum evaluation. J Med Educ. 1970;45(10):754-9. DOI: 10.1097/00001888-197010000-00006
91. *Rotthoff T, Ostapczuk MS, De Bruin J, Decking U, Schneider M, Ritz-Timme S. Assessing the learning environment of a faculty: psychometric validation of the German version of the Dundee Ready Education Environment Measure with students and teachers. Med Teach. 2011;33(11):e624-36. DOI: 10.3109/0142159X.2011.610841
92. Rotthoff T, Soboll S. Qualitätsverbesserung von MC Fragen: Ein exemplarischer Weg für eine medizinische Fakultät [Quality assurance of Multiple Choice Questions: an exemplary way for a medical faculty]. GMS Z Med Ausbild. 2006;23(3):Doc45. Available from: http://www.egms.de/en/journals/zma/2006-23/zma000264.shtml
93. Schmidt B, Loßnitzer T. Konzepte für fachinternes Qualitätsmanagement in der Lehre: Das Modell des Universitätsprojekts Lehrevaluation an der Friedrich-Schiller-Universität Jena. Qualität in der Wissenschaft. 2007;1(2):44-50.
94. *Schulze J, Drolshagen S. Format und Durchführung schriftlicher Prüfungen [Format and implementation of written assessments]. GMS Z Med Ausbild. 2006; 23(3):Doc44. Available from: http://www.egms.de/en/journals/zma/2006-23/zma000263.shtml

*=specific to medical education and cited in the article / medizinspezifisch und in dem Artikel zitiert
**=not specific to medical education and cited in the article / nicht-medizinspezifisch und in dem Artikel zitiert

95. Schulze J, Drolshagen S, Nürnberger F, Ochsendorf F, Schäfer V, Brandt C. Einfluss des Fragenformates in Multiple-choice-Prüfungen auf die Antwortwahrscheinlichkeit: Eine Untersuchung am Beispiel mikrobiologischer Fragen [The effects of phrasing on the answer likelihood in multiple choice based examinations: a case study in microbiology]. GMS Z Med Ausbild. 2005;22(4):Doc218. Available from: http://www.egms.de/en/journals/zma/2005-22/zma000218.shtml
96. Schuwirth LW, van der Vleuten CP. Changing education, changing assessment, changing research? Med Educ. 2004 Aug;38(8):805-12. DOI: 10.1111/j.1365-2929.2004.01851.x
97. Schuwirth LW, van der Vleuten CP. Different written assessment methods: what can be said about their strengths and weaknesses? Med Educ. 2004 Sep;38(9):974-9. DOI: 10.1111/j.1365-2929.2004.01916.x
98. Shea JA, Bellini LM. Evaluations of clinical faculty: the impact of level of learner and time of year. Teach Learn Med. 2002;14(2):87-91. DOI: 10.1207/S15328015TLM1402_04
99. *Soemantri D, Herrera C, Riquelme A. Measuring the educational environment in health professions studies: a systematic review. Med Teach. 2010;32(12):947-52. DOI: 10.3109/01421591003686229
100. **Sorenson DL, Johnson TD. Online student ratings of instruction. New Dir Teach Learn. 2003;2003(96):1-112.
101. Spiel C, Gössler PM. Zum Einfluß von Biasvariablen auf die Bewertung universitärer Lehre durch Studierende. Z Pädagog Psychol. 2000;14(1):38-47. DOI: 10.1024//1010-0652.14.1.38
102. *Spiel C, Schober B, Reimann R. Evaluation of curricula in higher education: challenges for evaluators. Eval Rev. 2006 Aug;30(4):430-50. DOI: 10.1177/0193841X05285077
103. Spiel C, Wolf P, Popper V. Lehre und Lehrevaluation – (un)geliebt? Z Psychol. 2002;210(1):27-39.DOI: 10.1026//0044-3409.210.1.27
104. Spooren P, Mortelmans D. Teacher professionalism and student evaluation of teaching: will better teachers receive higher ratings and will better students give higher ratings? Educ Stud. 2006;32(2):201-14. DOI: 10.1080/03055690600631101
105. *Stalmeijer RE, Dolmans DH, Wolfhagen IH, Muijtjens AM, Scherpbier AJ. The Maastricht Clinical Teaching Questionnaire (MCTQ) as a valid and reliable instrument for the evaluation of clinical teachers. Acad Med. 2010 Nov;85(11):1732-8. DOI: 10.1097/ACM.0b013e3181f554d6
106. Theisel N, Stosch C, Koebke J. Evaluationsbemühungen an den Medizinischen Fakultäten in Deutschland – Ergebnisse einer Umfrage. Med Ausbild. 2000;17()1):18-21. Available from: https://gesellschaft-medizinische-ausbildung.org/files/ZMA-Archiv/2000/1/Theisel_N,_Stosch_C,_Koebke_J.pdf
107. **Ting KF. A multilevel perspective on student ratings of instruction: Lessons from the Chinese experience. Res High Educ. 2000;41(5):637-61. DOI: 10.1023/A:1007075516271
108. van den Bussche H, Kreysing M. Leistungsorientierte Mittelvergabe in der Lehre an den medizinischen Fakultäten: Konzept und Praxis an der Hamburger Medizinischen Fakultät. Wissenschaftsmanagement. 2005;5:27-33. Available from: http://www.wissenschaftsmanagement.de/management/leistungsorientierte-mittelvergabe-der-lehre-den-medizinischen-fakultaeten

*=specific to medical education and cited in the article / medizinspezifisch und in dem Artikel zitiert
**=not specific to medical education and cited in the article / nicht-medizinspezifisch und in dem Artikel zitiert

109. *van den Bussche H, Weidtmann K, Kohler N, Frost M, Kaduszkiewicz H. Evaluation der ärztlichen Ausbildung: Methodische Probleme der Durchführung und der Interpretation von Ergebnissen [Evaluation of medical education: methodological problems of implementation and interpretation of results]. GMS Z Med Ausbild. 2006;23(2):Doc37. Available from: http://www.egms.de/en/journals/zma/2006-23/zma000256.shtml
110. van der Vleuten C. Validity of final examinations in undergraduate medical training. BMJ. 2000 Nov 11;321(7270):1217-9. DOI: 10.1136/bmj.321.7270.1217
111. van der Vleuten CP, van Luyk SJ, Beckers HJ. A written test as an alternative to performance testing. Med Educ. 1989;23(1):97-107. DOI: 10.1111/j.1365-2923.1989.tb00819.x
112. Weber A, Wacker A, Weltle D, Lehnert G. Stellenwert der Lehre an den deutschen medizinischen Fakultäten [Assessment of teaching at the faculties of medicine in Germany]. Dtsch Med Wochenschr. 2000 Dec;125(51-52):1560-4. DOI: 10.1055/s-2000-9525
113. Wenghofer E, Klass D, Abrahamowicz M, Dauphinee D, Jacques A, Smee S, Blackmore D, Winslade N, Reidel K, Bartman I, Tamblyn R. Doctor scores on national qualifying examinations predict quality of care in future practice. Med Educ. 2009 Dec;43(12):1166-73. DOI: 10.1111/j.1365-2923.2009.03534.x
114. Winkel O. Qualität statt Quantität Anreize zur Verbesserung der Lehre durch Teaching Points. Qualität in der Wissenschaft. 2008;2(4):86-8.
115. Woloschuk W, Coderre S, Wright B, McLaughlin K. What factors affect students' overall ratings of a course? Acad Med. 2011 May;86(5):640-3. DOI: 10.1097/ACM.0b013e318212c1b6
116. *Zuberi RW, Bordage G, Norman GR. Validation of the SETOC instrument – Student evaluation of teaching in outpatient clinics. Adv Health Sci Educ Theory Pract. 2007 Feb;12(1):55-69. DOI: 10.1007/s10459-005-2328-y

*=specific to medical education and cited in the article / medizinspezifisch und in dem Artikel zitiert
**=not specific to medical education and cited in the article / nicht-medizinspezifisch und in dem Artikel zitiert
